# Niche Variability and Its Consequences for Species Distribution Modeling

**DOI:** 10.1371/journal.pone.0044932

**Published:** 2012-09-10

**Authors:** Matt J. Michel, Jason H. Knouft

**Affiliations:** Department of Biology, Saint Louis University, St. Louis, Missouri, United States of America; National Institute of Water & Atmospheric Research, New Zealand

## Abstract

When species distribution models (SDMs) are used to predict how a species will respond to environmental change, an important assumption is that the environmental niche of the species is conserved over evolutionary time-scales. Empirical studies conducted at ecological time-scales, however, demonstrate that the niche of some species can vary in response to environmental change. We use habitat and locality data of five species of stream fishes collected across seasons to examine the effects of niche variability on the accuracy of projections from Maxent, a popular SDM. We then compare these predictions to those from an alternate method of creating SDM projections in which a transformation of the environmental data to similar scales is applied. The niche of each species varied to some degree in response to seasonal variation in environmental variables, with most species shifting habitat use in response to changes in canopy cover or flow rate. SDMs constructed from the original environmental data accurately predicted the occurrences of one species across all seasons and a subset of seasons for two other species. A similar result was found for SDMs constructed from the transformed environmental data. However, the transformed SDMs produced better models in ten of the 14 total SDMs, as judged by ratios of mean probability values at known presences to mean probability values at all other locations. Niche variability should be an important consideration when using SDMs to predict future distributions of species because of its prevalence among natural populations. The framework we present here may potentially improve these predictions by accounting for such variability.

## Introduction

Recent advances in the application of geographic information systems (GIS) analytical techniques have offered a powerful opportunity to predict species distributions in the context of spatially and temporally variable habitats [1], [2]. In particular, correlative species distribution modeling (SDM) techniques have applied a niche-based approach that identifies regions containing suitable environmental conditions based on habitat characteristics at locations of known species occurrences. Suitable areas can then be extrapolated onto other geographic regions or into the future using forecasted environmental conditions to predict a species' distribution [3], [4]. This method has proven useful for conservation planning by predicting the occurrence of rare species [5], responses of species to global climate change [6], and the impacts of invasive species [7]. However, predicting a species' response to habitat variability not only requires a quantitative framework that supports the prediction of a species' distributions in a spatial context, but also an understanding of how habitats change and whether a species' habitat preferences are conserved over space and time.

A fundamental assumption of the majority of SDMs, particularly those predicting species occurrences in temporally or spatially novel regions, is that the niche is conserved within a taxon [1], [8], otherwise known as niche conservatism [9]. From an evolutionary perspective, niche conservatism predicts that traits are retained to some degree in closely related taxa, with changes to the position of the niche defined as a ‘niche shift’ [9]. On ecological timescales the niche of some species has been shown to be quite flexible, such as for species colonizing new geographic areas [10], experiencing annual variation in climatic variables [11], or responding to seasonal variation [12]. Such niche variability has presented a challenge to the ability of correlative SDMs to predict changes in species' distributions in response to environmental change, to the extent that the failure of predictions from SDMs has been used as evidence for niche shifts [10].

**Figure 1:**
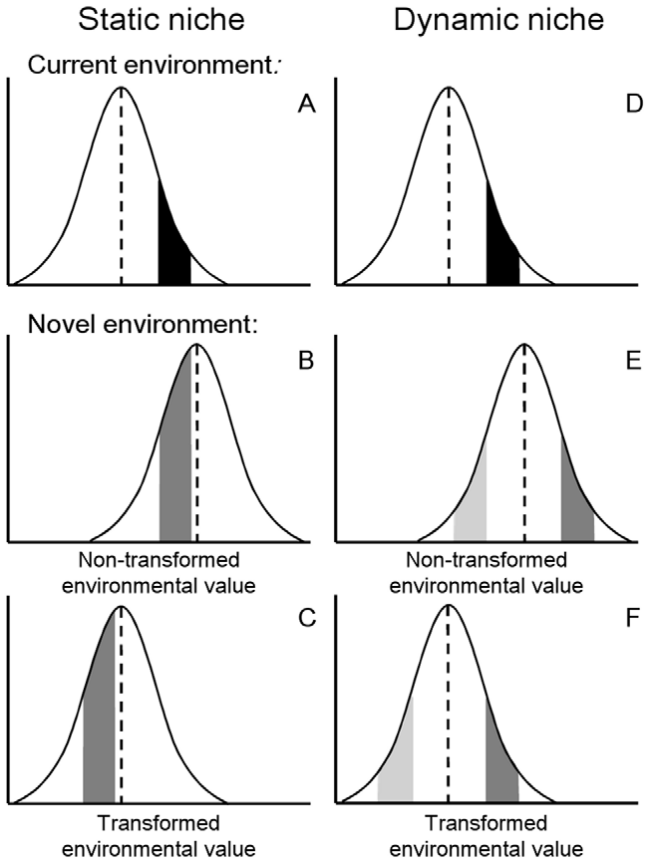
Consequences of niche variability for species distribution models. The curves represent frequency distributions of a hypothetical environmental variable. Black and gray shaded areas represent the current and projected range, respectively, of the environmental value which a species inhabits. The dashed line indicates the mean value of the environmental variable. A) and D) – current environmental conditions and species habitat use. B) – novel environmental conditions (frequency shifts to the right), and the response of a species whose niche is static. C) – the effect of a transformation of the environmental variable when a species' niche is static. E) – novel environmental conditions, and the response of a species that alters its niche and either maintains niche position (dark gray) or does not maintain niche position (light gray). F) – the effect of a transformation of the environmental variable when a species' niche is dynamic.

In response to environmental change, a species may either maintain the range of environmental conditions it inhabits (i.e., a static niche), or shift its habitat use relative to the distribution of the novel environmental conditions (i.e., a dynamic niche). When the niche is static, an SDM should accurately predict occurrences of that species in the novel environment because the habitats associated with the presence of the species do not change, regardless of changes in the available environment (Fig. 1A – B). However, when the niche is dynamic, an SDM would perform poorly, as it would predict occurrences in areas of the novel environment that would not be occupied by the species (Fig 1D – E). For niches that vary spatially or temporally, the new niche may retain the same position from the mean of the environmental variable (dark-shaded portion in Fig. 1E) if there is strong selection on niche traits as a result of directional environmental change [13] or a preference of the species to occupy, for example, the wettest microhabitats in a particular environment [11]. In such cases, a transformation that scales both the current and future environmental distributions to similar values (e.g., a *Z-*score standardization [14], or centering the means [15]) could align the distributions (Fig. 1F) and increase the accuracy of SDMs. However, this transformation would not improve the predictions for species with static niches (Fig. 1C), or for species with dynamic niches that do not maintain the niche position held in the original environment (light-shaded portion in Fig. 1E – F). Thus, proper forecasting of species distributions in novel environments may require a prior assessment of niche dynamics and a method to accommodate niche variability.

Whereas the vast majority of SDM studies have been conducted at broad geographic scales using relatively coarse climate and landscape environmental variables [16], [17], ongoing technical advances, particularly associated with spatial data acquisition, afford the opportunity to apply the niche-based SDM approach at smaller scales; for example, to predict the distribution of rare taxa at fine spatial scales [18]. An advantage of a local-scale SDM approach is the utilization of environmental data that are more specialized to particular taxonomic groups, rather than the potentially spurious environmental data (e.g., annual temperature, precipitation) that is prevalent in many broad-scale SDM studies. Knouft *et al*. [12] recently demonstrated the utility and benefit of applying GIS data to characterize the environmental niche and predict seasonal variation in population density among species in a fish assemblage of a local temperate stream. While [12] did not apply an SDM approach, the demonstrated intra-annual variability in stream habitat provides an opportunity to assess the accuracy of SDMs to predict species distributions in the face of environmental change.

The primary goal of this research is to determine if SDMs can accurately predict the distribution of a species with a variable niche. Specifically, we use the same dataset collected in [12] to address the following questions: 1) to what degree does the niche of stream fishes change seasonally and annually?; 2) does observed niche variability affect the accuracy of SDMs in predicting the local distribution of stream fishes?; and 3) can transformations of environmental variables improve the predictions from SDMs?

## Methods

### Ethics Statement

This research was approved by the Institutional Animal Care and Use Committee (Permit #2056). The Missouri Department of Natural Resources has issued permits for the collection of fish (year 2007: Permit #13478; year 2008: Permit #13845).

### Dataset

Detailed methods on the collection of the fish locality and habitat data can be found in [12] and Appendix S1. Fishes and habitat data were collected in Labarque Creek (average wetted width  = 4.1 m), a third order tributary in the Meramec River drainage in eastern Missouri, USA (38.4254° N, 90.6832° W). Sampling efforts were conducted across the same 675 m length of stream on five dates: June 30-July 2, 2007 (hereafter, July 2007); October 29–30, 2007 (October 2007); January 14–15, 2008 (January 2008); April 26–27, 2008 (April 2008); and July 7–8, 2008 (July 2008).

**Figure 2:**
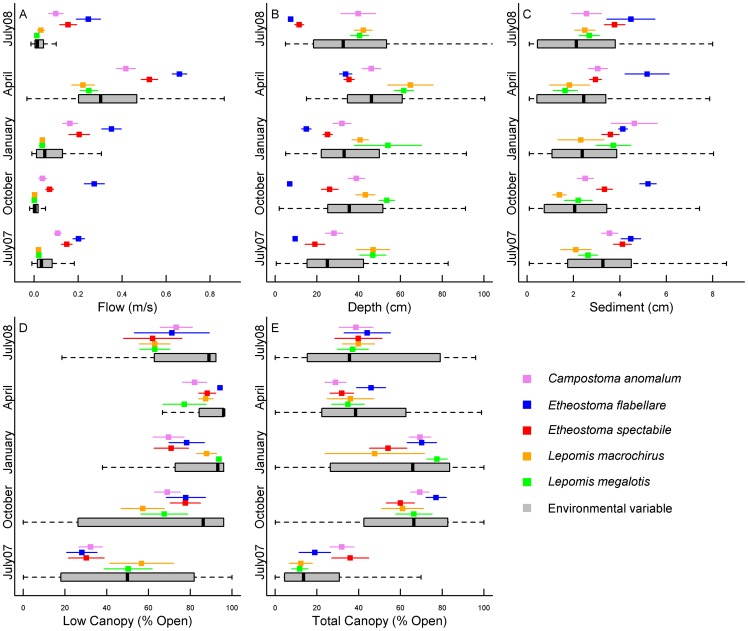
Niche dynamics of the five stream fish species. Each plot represents one habitat variable (represented in the plot by the gray boxplot; outliers not shown). A) flow rate (m/s), B) depth (cm), C) sediment size (cm), D) low canopy (% open) and E) total canopy (% open). Each color represents the mean habitat use (± 1 SE) of a fish species for that particular season (July 2007 through July 2008).

Fishes were collected with seine nets (1.2m×2.4m, 6.4 mm mesh) and a Smith-Root LR-20 backpack electrofisher (Smith-Root Inc., Vancouver, Washington, USA) during each sampling period. Individuals were identified to species and returned to the stream. While the dataset includes 11 species, we focused our analyses on five species: Central Stoneroller (*Campostoma anomalum*), Fantail Darter (*Etheostoma flabellare*), Orangethroat Darter (*E. spectabile*), Bluegill (*Lepomis macrochirus*), and Longear Sunfish (*L. megalotis*). These species were chosen because they were common and represented a wide range of habitat occupancy (e.g., *E. flabellare* is a riffle specialist while *L. megalotis* is a pool specialist; [12]; Fig. 2).

The day prior to fish sampling, data on five habitat variables were collected: flow rate (m/s; average of benthic, midwater, and surface rates), depth (cm), riparian vegetation cover less than three meters in height (% open; low canopy cover), total canopy openness (% open), and sediment size (cm). Georeferenced habitat localities from each sampling period were imported into ArcGIS, ver. 9.2. An inverse distance weighting (IDW) method using the three closest habitat measures was employed to generate 0.5 m resolution raster data layers for each of the seven habitat datasets [12]. These raster layers are necessary for the particular SDM used in this study (see *Methods – Species Distribution Modeling*).

### Estimating seasonal and annual niche dynamics

For each season, the georeferenced localities of the five fish species were intersected with each of the five habitat data layers to obtain a value of each environmental variable where the fish species occurred. Differences in habitat use between July 2007 and October 2007, January 2008 and April 2008 (i.e., seasonal niche dynamics) and July 2008 (i.e., annual niche dynamics) were tested using a separate multivariate analysis of variance (MANOVA) for each species (i.e. five MANOVAs) with season as the independent variable. For each species, a univariate analysis of variance (ANOVA) testing the effect of season on each environmental variable was conducted if significance was detected with the MANOVA. For any significant ANOVA, a multiple comparison test (false-discovery rate) was used to test the *a priori* null hypotheses that habitat use in October 2007, January 2008, April 2008 and July 2008 did not differ from July 2007. A dynamic niche was inferred if the habitat use for any environmental variable significantly differed from the habitat use of July 2007. We tested for differences in habitat use from July 2007, because this season served as our training data in the SDM (see *Methods – Species Distribution Modeling*).

A species may exhibit variability in the niche but not maintain niche position (see Figs. 1 E & F), defined as the distance between the mean value of the environment within the available habitat and the mean value of the environment inhabited by the species [19]. We tested for this possibility for all environmental variables to which species exhibited significance in the above multiple comparison tests. First, we aligned the environmental distributions of each season using a *Z*-score transformation [14]. Then, we used a *t*-test to determine if the distribution of environmental values inhabited by a species differed from July 2007 to a given season. If the null hypothesis was rejected, then it was concluded that the species altered its niche position as well as its niche. All statistical tests were conducted using R.2.9.2 [20].

**Table 1:** Accuracy of within-year and among-year projections from Maxent.

		Non-transformed Method				Transformed Method			
Date	*N*	*Presence*	*Other*	P-*value*	*Ratio*	*Presence*	*Other*	P-*value*	*Ratio*
*Campostoma anomalum*									
October 07	28	0.399	0.367	0.120	**1.033**	0.297	0.284	0.486	1.013
January 08	22	0.505	0.406	0.009*	**1.104**	0.393	0.360	0.118	1.033
April 08	23	0.478	0.493	0.770	0.981	0.394	0.373	0.258	**1.022**
July 08	16	0.409	0.362	0.113	1.048	0.448	0.386	0.086	**1.064**
*Etheostoma flabellare*									
October 07	9	0.476	0.042	<0.001*	1.544	0.597	0.057	<0.001*	**1.718**
January 08	14	0.316	0.056	<0.001*	1.297	0.529	0.126	<0.001*	**1.497**
April 08	16	0.026	0.006	0.008*	1.021	0.364	0.114	<0.001*	**1.284**
July 08	5	0.451	0.055	<0.001*	1.486	–	–	–	–
*Etheostoma spectabile*									
October 07	18	0.339	0.205	<0.001*	1.144	–	–	–	–
January 08	15	0.394	0.268	0.023	**1.135**	0.376	0.251	0.018	1.133
April 08	25	0.344	0.283	0.022	1.064	0.285	0.180	0.002*	**1.111**
July 08	9	0.426	0.214	0.003*	1.236	–	–	–	–
*Lepomis macrochirus*									
October 07	13	0.497	0.442	0.073	1.057	0.576	0.520	0.107	**1.058**
January 08	3	0.394	0.310	0.245	1.087	–	–	–	–
April 08	7	0.132	0.048	0.028	1.088	0.477	0.356	0.037	**1.129**
July 08	22	0.470	0.401	0.024*	1.071	–	–	–	–
*Lepomis megalotis*									
October 07	11	0.313	0.301	0.398	1.012	–	–	–	–
January 08	3	0.215	0.245	0.572	0.971	0.372	0.330	0.382	**1.042**
April 08	10	0.070	0.025	0.090	1.046	0.426	0.320	0.059	**1.113**
July 08	19	0.422	0.301	0.004*	**1.128**	0.526	0.421	<0.001*	1.111

*N* – number of localities for each species in each season. *Presence* and *Other* refer to the mean probability of presence at actual species occurrences and at all other locations, respectively. *P*-values generated from randomization procedures indicate whether the mean probability of occurrence differs from a random sample of localities. Non-transformed and Transformed method refer to species distribution models trained and projected using non-transformed environmental data or a mixed approach of non-transformed and *Z*-score transformed environmental data, respectively. *Ratio* refers to an evaluation of model performance calculated as the exponent of *Presence* divided by the exponent *Other*. Bold indicates that the ratio was greater for that particular method. Dashes indicate that test was not performed as the species during the given season did not significantly alter its niche. * – indicates significance based on a sequential Bonferroni correction (α′) when α = 0.05 (number of tests  = 5).

### Species distribution modeling

Using the locality data for each species and the GIS-based habitat data sets, we modeled each species' environmental niche using Maxent [21], because Maxent has been demonstrated to perform better than other commonly used SDMs when the sample size is small [22]. We chose the logistic output in Maxent, which generates a probability of occurrence map (ranging from 0 to 1.0) representing the likelihood that a species will occupy a particular site in the stream [21].

During model development, we trained each SDM on data collected in July 2007. Then, we projected this SDM onto the habitat data collected in October 2007, January 2008, April 2008 and July 2008 using one of two different methods. For the first method (hereafter, “non-transformed”), the SDM was trained on the raw habitat data from July 2007 and projected onto the raw habitat data from each season. This non-transformed approach thus represents the typical methodology in using SDMs to predict changes in a species' distribution. For the second method (hereafter, “transformed”), the SDM was trained and projected using a combination of non-transformed and transformed habitat data. If a species significantly varied its niche and maintained niche position for any particular season, the values of the environment to which the species altered its niche were first transformed using a *Z*-score transformation [14] in both training and projecting the SDM. Because this transformation would only apply if the species varied its niche (see Fig. 1C), the values of environments to which species did not alter its niche were not transformed.

**Figure 3:**
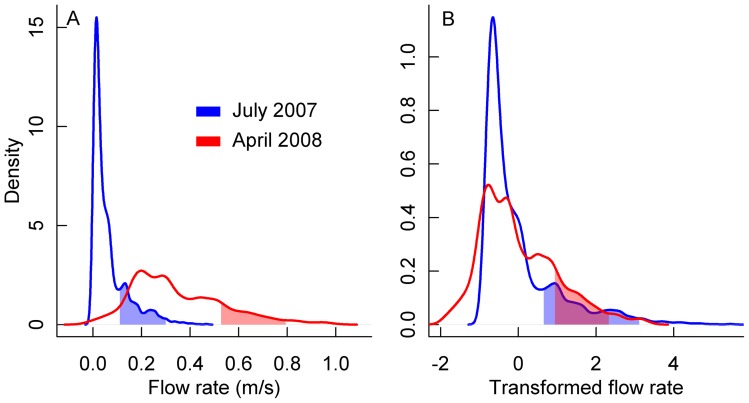
Demonstration of the effectiveness of the *Z*-score transformation. Plots are density functions of (A) non-transformed flow rate and (B) transformed flow rate in July 2007 and April 2008. Shaded portions are the mean ±1 SD of flow rate (m/s) of the locations in which the Fantail Darter, *Etheostoma flabellare*, occurred.

### Accuracy and comparison of the SDM projections

Our sampling approach allows us to directly test whether an SDM predicts species habitat use better than a random expectation (i.e., statistically reject the null hypothesis). However, because our samples are not independent in the sense that all the testing data originates from the same area, this test can only address the temporal transferability of the SDMs. To test our null hypothesis, we used a randomization procedure to determine if each species' localities (from a given collection period) were within areas with greater probability of occurrence values than other localities selected at random. As an example, a species' probability of occurrence map generated for July 2008 was intersected with collection locations from the July 2008 sampling period, and a mean probability of occurrence was obtained. This mean was then compared to a distribution of 1,000 mean probability values calculated from randomly selecting the same number of probability of occurrences as there were collection locations. A *P*-value was generated by dividing the rank of the initial mean probability of occurrence among this generated distribution by 1,000. A one-tailed test was used because we were testing if the initial mean probability of occurrence was significantly greater than the distribution of randomly generated mean probability values.

To directly compare the accuracy of the non-transformed and transformed methods, we computed a measure of model performance by dividing the mean of the probability of occurrences of the locations in which the species was observed (as above) by the mean of the probability of occurrence of all other locations. This ratio compares the probability of occurrences produced by Maxent at known species presences to the probability of occurrences produced by Maxent at all other locations. Thus, greater values of this ratio indicate models that produce greater probability values at known species occurrences relative to all other locations. We exponentially transformed each mean probability value before obtaining the ratio. This transformation provides a more appropriate weighting towards higher overall probability of occurrences, which is a priority of SDMs.

## Results

### Seasonal and annual niche dynamics

All environmental variables exhibited some degree of seasonal variability (see Fig. 2, Table S1, and [12]). In response to these seasonal changes, each species exhibited some degree of niche variability (Table S1; Fig. 2). More species altered habitat use in regard to changes in flow rate (5 of 5 species), water depth (4/5) and total canopy (4/5) than for low canopy (3/5) or sediment (1/5). Regarding seasonal niche dynamics, in October 2007 and January 2008, most species altered their niche in response to changes in total canopy (Oct – 4 species; Jan – 3 species) and low canopy (Oct – 3 species; Jan – 3 species), and in April 2008, most species altered their niche in response to flow (5 species) and water depth (3 species; Fig. 2). Regarding annual niche dynamics, *C. anomalum* significantly altered its niche in response to changes in low canopy cover, and *L. megalotis* significantly altered its niche in response to changes in total canopy cover (Fig. 2). Across all seasons, *C. anomalum*, *E. flabellare*, and *E. spectabile* exhibited the most niche variability, while *L. macrochirus* and *L. megalotis* exhibited the least (Table S2). There were two instances in which a species altered its niche, but did not maintain niche position: *E. spectabile* in response to changes in low canopy in October 2007 and *L. megalotis* in response to changes in total canopy in January 2008 (Fig. 2).

### Performance of species distribution models

Because the same hypothesis was tested for five different species within each season, α = 0.05 was modified using a sequential Bonferroni correction (α') to reduce the possibility of a Type I error. SDMs developed using the non-transformed method successfully predicted the occurrence of *E. flabellare* in all seasons, *E. spectabile* in October 2007 and July 2008, *C. anomalum* in January 2007, *L. macrochirus* in July 2008, and *L. megalotis* in July 2008 (Table 1). These results were generally consistent with the predictions from SDMs developed using the transformed method, except that *C. anomalum* was no longer successfully predicted, while *E. spectabile* was successfully predicted in April 2008 (Table 1).

Ratios of the mean probability at occurrence sites to the mean probability at all other sites were computed to directly compare the predictions of the non-transformed and transformed SDM projection methods. Generally, model performance was greater for SDMs developed using the transformed method than SDMs developed using the non-transformed method (Table 1). Of the 14 projections made using both methods (for species that either did not significantly alter their niche or did alter their niche but did not maintain niche position, the projections would be the same under both methods), the transformed method produced greater ratios in ten of the cases.

## Discussion

Niche conservatism as applied in GIS-based studies is commonly viewed as an evolutionary phenomenon [9]. In local assemblages, the consistency of niche characteristics within and among years over relatively short time spans (e.g., within generations) can most parsimoniously be attributed to the overall range of habitat that is suitable for a species compared to the actual habitat that is available during various times of the year (i.e., a plastic response to variation in available habitat). From this perspective, a species that can tolerate a relatively narrow range of habitats would be constrained to similar types of habitats throughout the year, whereas a species that can tolerate a relatively wide range of habitats may exhibit habitat shifts in response to seasonal variation. In this study, we find that these niche shifts are prevalent among five species of stream fishes and can affect SDM predictions. Additionally, we demonstrate that a simple transformation of environmental variables can account for niche shifts and improve projections from SDMs.

Each of the five stream fish species significantly altered their niche across the sampling periods, mostly in response to variation in flow rate, total canopy and low canopy. Such seasonal variation in microhabitat use among stream fishes appears to be common [23]–[25], especially in response to flow variability [26]. Our study suggests that stream fishes tend to shift habitat use with respect to flow, regardless of the overall distribution of flow rates within a stream (i.e., fish found in low-flow microhabitats should always be found in areas with the lowest flow if some environmental change increases the overall flow rates in the stream). In contrast, we found that stream fishes seem to have strong preferences for sediment size, as it was the only environmental variable to which fish did not significantly alter their niche. Thus, this study suggests that fishes may be able to shift habitat use in response to changes in flow rates, but not sediment size. Consequently, a change in the flow regime of a stream may only result in the habitat shifts of species, whereas a change to the substrate of a stream (e.g. by sedimentation) may result in the local extirpations of certain species. Because hydrologic changes in streams often promote changes to substrate, our research suggests that the mechanism underlying any subsequent alterations to fish assemblages may be changes to substrate rather than flow.

The degree to which species altered their niches differed, as the two sunfishes (*L. macrochirus* and *L. megalotis*) exhibited the most static niches, while a minnow (*C. anomalum*) and two darters (*E. flabellare* and *E. spectabile*) exhibited the most dynamic niches. These patterns are similar to a previous study, which found that sunfish (Centrarchidae) were more consistent in habitat use across seasons than minnows (Cyprinidae) [25]. These species-specific and habitat-specific differences in niche variability suggest that a thorough examination of a species' niche dynamics is recommended before SDMs are used to predict distributions under future environmental change or in new geographic areas.

Niche variability appeared to affect the ability of SDMs to predict future distributions. The transformed method which was designed to take into account niche variability produced greater accuracy ratios for ten of 14 projections (Table 1). This improvement was most notable for the April 2008 projections, in which the transformed method performed substantially better for each of the five species. In April 2008, mean overall flow rate of the stream increased from 0.064 m/s to 0.347 m/s. In response to this increase, species maintained their niche position relative to the mean overall flow rate (see Fig. 3 for an example with *E. flabellare*). An SDM trained and projected using the non-transformed data would be projecting onto novel environmental space, sometimes termed the ‘problem of non-analog climate’ [27]. Typically, SDMs, such as Maxent, recognize these novel conditions as at the limit of the training data, which can lead to an underestimate of a species' true probability of occurrence (see the mean probability of occurrence values for *E. flabellare* in April 2008 using the non-transformed method in Table 1). However, by standardizing the environmental values for both sampling periods, the problem of novel environmental conditions is minimized, as the range of the training data coincides with the range of the projection data (Fig. 3). Although we used a presence-only SDM, this method of transforming environmental data would be appropriate for presence-absence SDMs, such as generalized additive models and boosted regression trees.

Our results suggest that the SDM approach can make accurate predictions at local scales. With the exception of *C. anomalum*, our local-scale SDMs developed with July 2007 habitat and fish locality data were able to accurately predict species occurrences in July 2008 (Table 1). The species used in this study vary in their microhabitat use: *L. macrochirus* and *L. megalotis* specialize on pool habitats, *E. flabellare* and *E. spectabile* specialize on riffle habitats, while *C. anomalum* is ubiquitous in Labarque Creek exhibiting the most generalized habitat use of any fish species in the stream [12]. The poor fit for the generalist species, *C. anomalum*, is expected given previous research which indicates that SDM predictions tend to be more accurate for habitat specialists compared to generalists [28]. The resolution of GIS-based environmental data used in SDM studies is increasing [18], offering the opportunity to understand factors regulating species distributions at local scales using taxonomically appropriate environmental data. We expect this trend to continue, particularly considering the increased availability of high resolution remote sensing data (e.g., <10 m resolution [29]), and the ongoing need to understand species response to habitat variability at local scales as well as across scales. Applications at local scales potentially offer the opportunity to address basic questions regarding population dynamics and local community structure as well as more applied issues regarding species response to habitat modification and the distribution and impact of invasive species.

The possibility of a species' shift in habitat use should be an important consideration when projecting SDMs in novel environmental conditions. Our research focused on seasonal niche variation, but other potential examples of niche variability may be evident in species colonizing new habitats [10] or responding to anthropogenic alterations to habitat such as global climate change [30]. Here, we provide a framework for assessing the niche dynamics of species in response to environmental change and adjusting environmental data of SDMs to take into account any observed variation in the niche. This method of standardizing environmental data could expand the applicability of SDMs for species with flexible niches or species inhabiting variable environments. Additionally, this standardization of environmental data could also permit an analysis of the distribution of a species if its niche adaptively evolved in response to changing environmental conditions [13]. We expect that further research on the niche shifts of different species in response to various environmental conditions will not only improve the projections of SDMs, but also identify the environmental variables most likely to induce changes to a species' distribution.

## Supporting Information

Appendix S1 **Detailed methods on data collection.**
(DOCX)

Table S1 **Niche variability for the five species of stream fishes.**
(DOCX)

Table S2 **Environmental variables to which each of the five species of stream fishes altered their respective niche.**
(DOCX)
