# Supplementary material for: Niche Variability and Its Consequences for Species Distribution Modeling
Source: PLoS One. 2012 Sep 10;7(9):e44932. doi: 10.1371/journal.pone.0044932 (PMC3438174; doi:10.1371/journal.pone.0044932)
Supplement: Appendix S1 — Detailed methods on data collection. (DOCX) [file pone.0044932.s001.docx]

**Appendix S1.** Detailed methods on data collection.

The dataset originates from [1], which contains further details on methodology. Here, we summarize the collection of fish localities and habitat data.

*Fish localities*

Sampling of fishes proceeded in an upstream direction starting at the downstream edge of the stream reach. Homogeneous microhabitats (i.e., riffle, run, pool) were isolated with block nets and physical habitat and sampled with seine nets (1.2m x 2.4m, 6.4mm mesh) and a Smith-Root LR-20 backpack electrofisher (Smith-Root Inc., Vancouver, Washington, USA). Microhabitats were approached slowly as to minimize behavioral disturbances to fishes. Additionally, sampled microhabitats were not contiguous, as to further minimize disturbance to upstream fish assemblages caused by sampling procedures conducted downstream. As all microhabitats were sampled to near-depletion, the time and number of seine hauls at each microhabitat varied, mostly by the number of individuals caught. Because these microhabitats varied across seasons, the location and number of fish sampling locations differed by sampling period: July 2007 (60 sites), October 2007 (56), January 2008 (46), April 2008 (56) and July 2008 (50). Collection locations were georeferenced with a Trimble GeoXH GPS unit (Trimble Navigation, Sunnyvale, California, USA), which provides sub-30 cm accuracy, and individual fishes were identified to species and returned to the stream.

Across all sampling seasons, 25 fish species were identified (see below for a list of all 25 species). Of these, 11 species were present during each sampling season, and we focused our analyses on five of these 11 species. These species were chosen because they were common (i.e., provided enough collection localities to train and test species distribution models), represented the most common taxonomic groups in the stream [minnows (Cyprinidae), darters (Percidae), and sunfish (Centrarchidae)], occupy different microhabitats (i.e., *E. flabellare* is a riffle-specialist and *L. megalotis* is a pool-specialist), and utilize different occupancy strategies (i.e., *C. anomalum* is a habitat generalist and *E. flabellare* is a habitat specialist).

*Habitat Data*

Local fish presence or absence is primarily determined by habitat variables [2]. Knouft *et al.* [1] found that the environmental niche of stream fishes in Labarque Creek is strongly shaped by a variety of habitat variables including benthic flow rate (m/s), midwater flow rate (m/s), surface flow rate (m/s), depth (cm), riparian vegetation cover less than three meters in height (% cover; low canopy cover), total canopy openness (% openness), and sediment size (mm). Some environmental variables (e.g., pH and dO_2_) were not measured because, although they have been shown to structure fish assemblages in other studies, they do not vary spatially over the small scales considered in this study.

Data on flow, depth, canopy cover and sediment were collected at georeferenced locations within the stream reach during each sample period on the day preceding fish sampling. The number of habitat sample locations ranged from 83 to 244 depending on the season and habitat variable. Habitat data were collected as in [1]: benthic flow rate was measured 2 cm above the substrate, midwater flow rate was measured in the middle of the water column, and surface flow rate was measured 2 cm below the surface with a Marsh-McBirney Flo-mate flow meter (Hach Company, Loveland, Colorado, USA); low canopy and total canopy were estimated with a spherical densitometer; and the maximum width of five randomly chosen pieces of substrate was measured within 1 m^2^ of the georeferenced habitat data collection point and used to calculate mean sediment size.

Georeferenced habitat datasets from each sampling period were imported into ArcGIS, ver. 9.2. An inverse distance weighting (IDW) method using the three closest habitat measures was employed to generate 0.5 m resolution raster data layers for each of the seven habitat datasets. Knouft *et al.* [1] demonstrated that the IDW approach using the closest three habitat measures is preferable to other interpolation options (i.e., kriging) in this system. Benthic, midwater, and surface flow rates were highly correlated. Thus, the average of these three variables was used as the estimate of flow rate, resulting in five GIS-based habitat variables for each sampling period (depth, flow rate, sediment size, low canopy, and total canopy).

List of fish species collected in Labarque Creek from July 2007 through July 2008. Bold indicates species used for analyses. Asterisks indicate species found in all sampling seasons.

| **Family** | **Scientific name** |
| --- | --- |
| Catostomidae | *Moxostoma duquesneii* |
| Centrarchidae | *Lepomis cyanellus* |
|  | *Lepomis humilis* |
|  | ***Lepomis macrochirus**** |
|  | ***Lepomis megalotis**** |
|  | *Micropterus punctulatus* |
| Cottidae | *Cottus bairdi* |
| Cyprinidae | ***Campostoma anomalum**** |
|  | *Ericymba buccata** |
|  | *Luxilus chrysocephalus** |
|  | *Luxilus zonatus* |
|  | *Lythrurus umbratilis** |
|  | *Notropis atherinoides* |
|  | *Pimpehales notatus* |
|  | *Semotilus atromaculatus** |
| Fundulidae | *Fundulus catenatus** |
|  | *Fundulus olivaceus** |
| Ictaluridae | *Ameiurus natalis* |
|  | *Noturus gyrinus* |
| Percidae | *Etheostoma blennioides* |
|  | *Etheostoma caeruleum* |
|  | ***Etheostoma flabellare**** |
|  | *Etheostoma nigrum* |
|  | ***Etheostoma spectabile**** |
| Poeciliidae | *Gambusia affinis* |

**References**

1. Knouft JH, Caruso NM, Dupre PJ, Anderson KR, Trumbo DR, et al. (2011) Using fine-scale GIS data to assess the relationship between intra-annual environmental niche variability and population density in a local stream fish assemblage. Methods Ecol Evol 2: 303-311.

2. Matthews WJ (1998) Patterns in Freshwater Fish Ecology. New York: Chapman and Hall. 784 p.
