# Supplementary material for: Niche Variability and Its Consequences for Species Distribution Modeling
Source: PLoS One. 2012 Sep 10;7(9):e44932. doi: 10.1371/journal.pone.0044932 (PMC3438174; doi:10.1371/journal.pone.0044932)
Supplement: Table S1 — Niche variability for the five species of stream fishes. (DOCX) [file pone.0044932.s002.docx]

**Table S1**. Niche variability for the five species of stream fishes. A) Results of multivariate analysis of variance on the effects of season on habitat use for each species. B) Results of subsequent univariate analysis of variance tests indicating the environmental variables to which each species altered its niche.

| A) Multivariate statistics for each species | | | | | | | | |
| --- | --- | --- | --- | --- | --- | --- | --- | --- |
| **Species** | **df** | | **Pillai Trace** | | ***F*** | | ***P*** | |
| *C. anomalum* | 4 | | 1.25 | | 10.4 | | **<0.001** | |
| *E. flabellare* | 4 | | 1.46 | | 5.76 | | **<0.001** | |
| *E. spectabile* | 4 | | 1.21 | | 6.77 | | **<0.001** | |
| *L. macrochirus* | 4 | | 1.02 | | 3.21 | | **<0.001** | |
| *L. megalotis* | 4 | | 1.37 | | 5.20 | | **<0.001** | |
|  |  | |  | |  | |  | |
| B) Univariate statistics (*F*-ratio and *P*-value) for each species by environmental variable | | | | | | | | |
| **Species** | **Flow** | **Total canopy** | | **Low canopy** | | **Sediment** | | **Depth** |
| *C. anomalum* | **26.3 (<0.001)** | **10.3 (<0.001)** | | **9.13 (<0.001)** | | **2.78 (0.030)** | | **3.23 (0.015)** |
| *E. flabellare* | **23.9 (<0.001)** | **4.52 (0.003)** | | **6.14 (<0.001)** | | 0.61 (0.660) | | **30.3 (<0.001)** |
| *E. spectabile* | **32.9 (<0.001)** | 2.35 (0.061) | | **7.95 (<0.001)** | | 1.61 (0.181) | | **10.4 (<0.001)** |
| *L. macrochirus* | **13.0 (<0.001)** | **3.25 (0.019)** | | 1.10 (0.369) | | 0.56 (0.690) | | 1.18 (0.331) |
| *L. megalotis* | **37.7 (<0.001)** | **8.05 (<0.001)** | | 1.66 (0.173) | | 1.15 (0.344) | | **2.78 (0.036)** |

Note: Bold indicates significance at α = 0.05
