# Supplementary material for: Niche Variability and Its Consequences for Species Distribution Modeling
Source: PLoS One. 2012 Sep 10;7(9):e44932. doi: 10.1371/journal.pone.0044932 (PMC3438174; doi:10.1371/journal.pone.0044932)
Supplement: Table S2 — Environmental variables to which each of the five species of stream fishes altered their respective niche. (DOCX) [file pone.0044932.s003.docx]

**Table S2**. Environmental variables to which each of the five species of stream fishes altered their respective niche. *P*-values are calculated from a false discovery rate multiple comparisons test comparing each sampling date to the July 2007 data.

|  | October 2007 | | January 2008 | | April 2008 | | July 2008 | |
| --- | --- | --- | --- | --- | --- | --- | --- | --- |
| Species | *Variable* | P*-value* | *Variable* | P*-value* | *Variable* | P*-value* | *Variable* | P*-value* |
| *C. anomalum* | low canopy  total canopy | <0.001  <0.001 | low canopy  total canopy | <0.001  <0.001 | depth  flow  low canopy | 0.008  <0.001  <0.001 | low canopy | <0.001 |
| *E. flabellare* | low canopy  total canopy | 0.005  0.009 | depth  flow  low canopy  total canopy | 0.037  0.012  0.005  0.009 | depth  flow  low canopy | <0.001  <0.001  <0.001 | (none) |  |
| *E. spectabile* | low canopy* | 0.001 | depth  low canopy | 0.029  0.001 | depth  flow  low canopy | <0.001  <0.001  <0.001 | (none) |  |
| *L. macrochirus* | total canopy | 0.012 | (none) | -- | flow | <0.001 | (none) |  |
| *L. megalotis* | total canopy | <0.001 | total canopy* | 0.004 | flow | <0.001 | total canopy | 0.041 |

Note: Asterisks (*) indicate environmental variables to which species exhibited niche plasticity, but did not maintain niche position.
